# Pharmacological Evaluation and Antifertility Activity of *Jatropha gossypifolia* in Rats

**DOI:** 10.1155/2013/125980

**Published:** 2013-10-08

**Authors:** Sachin Jain, Gajendra Pratap Choudhary, Dinesh Kumar Jain

**Affiliations:** ^1^Department of Pharmacognosy, College of Pharmacy, IPS Academy, AB Road, Indore, Madhya Pradesh 452012, India; ^2^School of Pharmacy, Devi Ahilya Vishwavidyalaya, Indore, Madhya Pradesh 452012, India

## Abstract

*Objectives*. Pharmacological and antifertility activity evaluation of *Jatropha gossypifolia* in rats. *Methods*. The antifertility activity of the extracts of *Jatropha gossypifolia* in rats was evaluated using two experimental animal models. Estrogenic activity was evaluated in immature female rats using ethinyl estradiol as standard. Anti-implantation and early abortifacient activity was performed in female Wistar rats by determining the number of implantations and implantation resorptions. *Results*. In estrogenic activity evaluations, the ethanolic and aqueous extracts offered significant estrogen-like activity at 400 mg kg^−1^ p.o. by increasing the uterine weight compared to vehicle control group. Ethanolic extract (400 mg kg^−1^, p.o.) treatment significantly decreased the number of implants and increased the number of resorptions compared to vehicle control group. *Conclusion*. The results of the present study provide the evidence of the anti-fertility activity of *Jatropha gossypifolia* as claimed in the traditional use. The results are consistent with the literature reports related to the antifertility effect of flower extracts of *Jatropha gossypifolia*.

## 1. Introduction

*Jatropha gossypifolia* (*JG*) (Euphorbiaceae), a common garden plant in tropical countries, has been used as a traditional medicine. Plants are well known as a major source of modern medicines. From ancient times, humans have utilized plants for the treatment or prevention of diseases, leading to the dawn of traditional medicine. *Jatropha gossypifolia *is one of the plants that are used in Chinese, Ayurvedic, and Thai traditional medicine for the treatment of fever, pain, and dysentery.

Population explosion has created a grave setback in the economic growth and all-round human development in developing countries. Current pandemic population explosion demands an immediate betterment of new potential contraceptives [1]. Studies of many years have highlighted the unmet demand for safe, inexpensive, and acceptable contraceptives to avoid unwanted pregnancies and resultant abortions. The quest for the oral contraceptive agent that can control human fertility is as old as recorded history. Although a wide variety of synthetic contraceptive agents [2, 3] are available, these cannot be used continuously due to their severe side effects [4, 5]. Hence, people are looking back to age-old tradition of using herbal medicines, which have minimum side effects. India in general and Western Ghats region in particular have enormous wealth of medicinal plants. Roots are used in leprosy, decoction of leaves is used as purgative and stomachic, leaves are used as febrifuge for intermittent fevers, and latex is used on ulcers. It contains compounds other than diterpenoids. The chief compounds reported are triterpenoid, sterol, alcohol, and hydrocarbon. The phenolic compounds include flavonoid lignans, coumarin tannin, phenanthrenes, quinones, phenolic acid, alkaloids, cyanogenic glycosides, and glucosinolates.

A literature survey reveals that no systematic approach has been made to study the reproductive toxicity of leaves of this plant. In the present work, we have investigated the reproductive toxicity of the ethanolic extract of *J. gossypifolia* leaves against ethinyl estradiol.

## 2. Materials and Methods

### 2.1. Animals

Female Swiss albino mice (18–22 g), Wistar albino rats 150–200 g, and immature female Wistar albino rats of 21–23 days old (40–60 g) were used in this study. They were procured from animal house, College of Pharmacy, IPS Academy, Indore, MP. The animals were acclimatized for ten days under laboratory conditions. They were housed in polypropylene cages and maintained at 27°C ± 2°C, relative humidity 65 ± 10% under a 12-hour light/dark cycle. The animals were fed with rodent pellet diet and water ad libitum. Animal ethical clearance for performing the experiments on animals was obtained from the Institutional Animal Ethical Committee (IAEC).

Each experimental group had a separate set of animals and care was taken to ensure that animals used for one response were not employed elsewhere. Animals were habituated to laboratory conditions for 48 h prior to experimental protocol which minimizes any nonspecific stress.

### 2.2. Plant Material

The leaves of *JG* were collected from Indore, Madhya Pradesh. The authentication was done by Professor S. R. Upadhayaya, Department of Botany, Govt. PG College Indore. A voucher specimen (COPIPS/T/098/2010) was deposited in the department.

### 2.3. Preparation of Extracts

The leaves of *Jatropha gossypifolia* were air-dried under shade, pulverized by a mechanical grinder, passed through 40 mesh and then stored in airtight containers. The powdered leaves were extracted with petroleum ether, ethanol (80% w/v), and distilled water using soxhlet extractor. These extracts were concentrated to dryness under reduced pressure and controlled temperature to yield solid masses that were completely free from solvents; the percentage yield of petroleum ether and ethanolic and aqueous extracts was found to be 2.8%, 48%, and 6% W/W, respectively.

### 2.4. Preliminary Phytochemical Screening

The preliminary phytochemical screening was carried out on petroleum ether, ethanol, and aqueous extracts of *Jatropha gossypifolia* leaves for the detection of various phytochemicals. Tests for common phytochemicals were carried out by standard methods described in Practical Pharmacognosy by Kokate and Khandelwal [6, 7].

### 2.5. Acute Toxicity Study

The acute toxicity for ethanol and aqueous extracts of *Jatropha gossypifolia* was determined in albino mice, maintained under standard conditions. The animals fasted overnight prior to the experiment, and fixed dose method was adopted as per OECD Guideline no. 420—fixed dose method.

### 2.6. Antifertility Activity

#### 2.6.1. Estrogenic Activity in Immature Female Rats

Immature female rats of Wistar strain 21–23 days old and weighing 40–60 g were used. They were divided into six groups of six animals each. The various groups were treated as follows:

 Group I: control (saline solution) p.o.,
 Group II: reference standard (ethinyl estradiol 0.02 mg kg^−1^, p.o.),
 Group III: ethanolic leaves extract of *JG* (200 mg kg^−1^, p.o.),
 Group IV: ethanolic leaves extract of *JG* (400 mg kg^−1^, p.o.),
 Group V: aqueous leaves extract of *JG* (200 mg kg^−1^, p.o.),
 Group VI: aqueous leaves extract of *JG* (400 mg kg^−1^, p.o.).

The treatment was given for six days, 24 h after the last treatment; all the animals were sacrificed by decapitation, and uteri were dissected out, cleared off the adhesive tissue, blotted on filter paper, and weighed quickly on a sensitive balance. The tissues were fixed in Bouin's fixative for 24 h, dehydrated in alcohol, and embedded in paraffin. The paraffin blocks were sectioned at 6 *μ* and stained with hematoxylineosin solution (H and E Stain) for histological observations [8, 9].

#### 2.6.2. Anti-Implantation Activity

Female rats of proestrus phase were kept with male rats of proven fertility in the ratio of 2 : 1. The female rats were examined in the following morning for evidence of copulation. The animal were which showed thick clumps of spermatozoa in vaginal smear was separated from the male partner. Only the rats with normal estrous cycles were selected for the experiment. The animals divided into six groups of six animals each. The various groups were treated as follows:

 Group I: control (saline solution) p.o.,
 Group II: reference standard (ethinyl estradiol 0.02 mg kg^−1^, p.o.),
 Group III: ethanolic leaves extract of *JG* (200 mg kg^−1^, p.o.),
 Group IV: ethanolic leaves extract of *JG* (400 mg kg^−1^, p.o.),
 Group V: aqueous leaves extract of *JG* (200 mg kg^−1^, p.o.),
 Group VI: aqueous leaves extract of *JG* (400 mg kg^−1^, p.o.).

The extracts were administered orally from day 1 t day 7 of gestation. On the 10th day, laparotomy was carried out under light ether anesthesia in sterile conditions. The uteri were examined to determine the number of implantation sites; the numbers of corpora lutea in ovaries were recorded. The abdomen was sutured, and the animals were left in cages. The drugs were administered orally again for 3 days (days 14–16). On the 18th day, laparotomy was carried out again for evaluating the early abortifacient activity. The percentages of anti-implantation activity were calculated using the formula given in

(1) %  Anti-implantation  activity  =100−No.  of  ImplantationsNo.  of  Corpora  lutea×100.

### 2.7. Statistical Analysis

Values were expressed as *x* ± *s* from 6 animals. Statistical difference in the mean analyzed using one-way ANOVA followed by Turkey's multiple comparison tests *P* < 0.05 was considered as statistically significant.

## 3. Results

### 3.1. Phytochemical Analysis

Preliminary phytochemical analysis of extracts revealed the presence of carbohydrates, steroids, glycosides, flavonoids, tannins, and alkaloids in both ethanolic and aqueous extract.

### 3.2. Acute Toxicity Study

No morbidity and mortality were detected till 2000 mg kg^−1^, p.o. for both ethanol and aqueous extracts; hence, ethanol and aqueous extracts were considered to be safe till 2000 mg kg^−1^, p.o. 

### 3.3. Antifertility Activity

#### 3.3.1. Estrogenic Activity on Immature Female Rats

**Figure 1:**
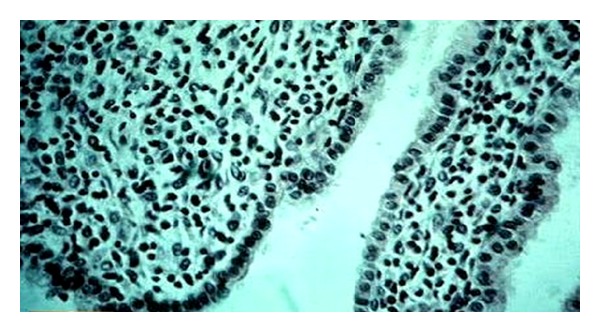
Photomicrograph showing section of uterus indicating surface epithelium with no secretary activity (control group) HE 300x.

**Figure 2:**
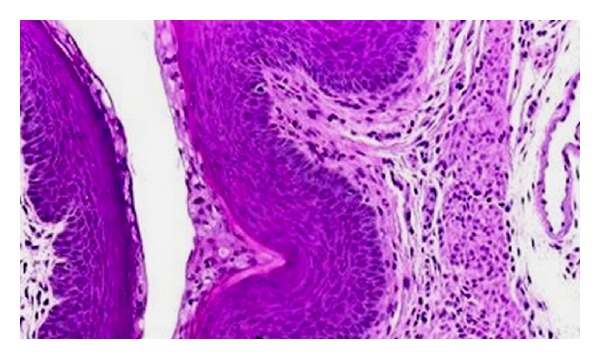
Photomicrograph showing section of uterus indicating increasing height of luminal epithelium (Ethinyl estradiol) HE 300x.

**Figure 3:**
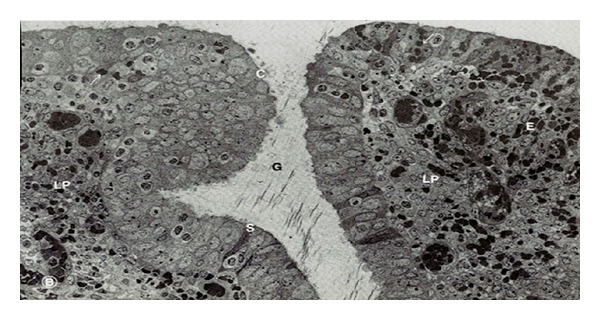
Photomicrograph showing section of uterus indicating increase in height of luminal epithelium (ethanolic extract: 200 mg kg^−1^) HE 300x.

**Figure 4:**
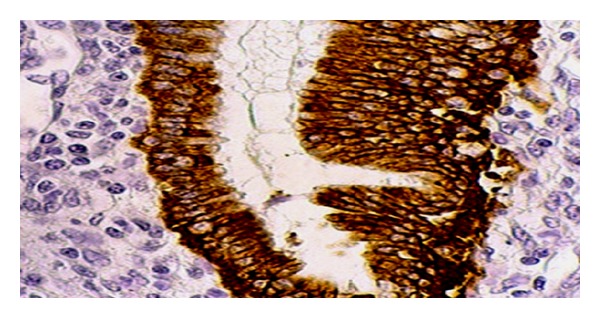
Photomicrograph showing section of uterus indicating increase in height of luminal epithelium and loose and edematous stroma with stimulated uterine glands (ethanolic extract: 400 mg kg^−1^) HE 300x.

**Figure 5:**
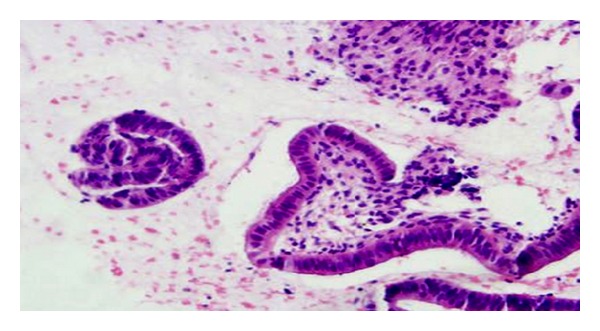
Photomicrograph shows section of uterus indicating moderate increase in height of luminal epithelium with moderate stimulation of uterine weight (aqueous extract: 200 mg kg^−1^) HE 300x.

**Figure 6:**
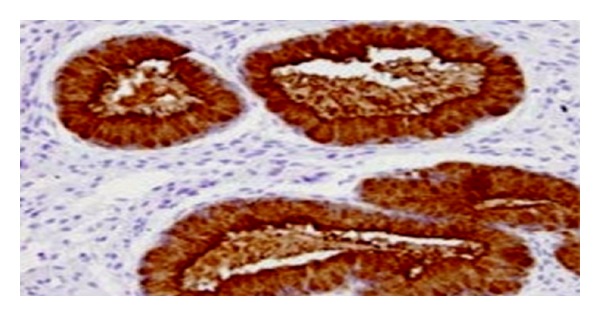
Photomicrograph showing section of uterus indicating moderate increase in height of luminal epithelium with stimulated uterine glands (aqueous extract: 400 mg kg^−1^) HE 300x.

Treatment with ethanolic (200 and 400 mg kg^−1^, p.o.) and aqueous extracts (200 and 400 mg kg^−1^, p.o.) had showed significant increase in uterine weight in a dose-dependent manner compared to vehicle control. The estrogenic effect of aqueous extract at 400 mg kg^−1^ p.o. was comparable with reference standard ethinyl estradiol (0.02 mg kg^−1^, p.o.). Furthermore, the ethanolic extract at 400 mg kg^−1^ offered more potent estrogenic activity than the reference standard ethinyl estradiol. The extract significantly increased the weights of uteri (Table 1), and results obtained were also correlated and supported by the histopathological findings, where the ethanolic extract (400 mg kg^−1^, p.o.) showed significant increase in the height of luminal epithelium and loose and edematous stroma with stimulated uterine glands, while the aqueous extract (400 mg kg^−1^, p.o.) showed moderate increase in the height of luminal epithelium with stimulated uterine glands (Figures 1, 2, 3, 4, 5, and 6).

#### 3.3.2. Anti-Implantation Activity

The anti-implantation activity is expressed as the percentage decrease in the number of implantations in the uteri on day 10 of pregnancy, and the number of resorbed implants from the existing number of implants will be recorded on day 18 for evaluating the early abortifacient activity. The ethanolic and aqueous extracts have offered significant and dependent anti-implantation and early abortifacient activity by decreasing the number of implantation sites and showed significant resorption of the existing implants compared to vehicle control. The ethanolic extract at 400 mg kg^−1^ p.o. showed 74.27% antifertility activity and it was found to be more potent than aqueous extract; at 400 mg kg^−1^ p.o., aqueous extract (400 mg kg^−1^ p.o.) offered 46.78% antifertility activity. The results are shown in Table 2.

The results obtained with the extracts on uterine weight (Table 1) of immature female rats were also supported by histological architecture. Histopathological studies were performed to know changes that occurred with extracts treatment. The section of control group shows normal architecture of uterus. It indicates surface epithelium with no secretary activity (Figure 1). Ethanolic extracts-treated groups have shown increase in height of luminal epithelium and loose and edematous stroma with stimulated uterine glands (Figures 1–6).

## 4. Discussion

It appears that ethanolic extracts have estrogenic activity at the dose of 400 mg kg^−1^ body weight as evident from the significant increase in the diameter of the uterus, height of the endometrial epithelium, and thickness of endometrium in extract-treated animals compared with control, while the animals treated with aqueous extract (400 mg kg^−1^ p.o.) showed increased height of luminal epithelium with stimulated uterine glands. These extracts did not exhibit any antiestrogenic activity. Proper equilibrium between estrogen and progesterone is essential for implantation, and any disturbance in the level of these hormones may affect the fertility [10].

Hence, the antifertility activity of the ethanolic and aqueous extracts may be mainly due to their estrogenic activity. The phytochemical constituents such as isoflavones along with coumestans (also flavonoids) and lignans belong to a class of substances known as nonsteroidal phytoestrogens, and they produce infertility in animals. In addition, it has also been proved that several commonly occurring flavonoids mimic the biological effects of 17**β*-*estradiol by virtue of their ability to bind and activate the nuclear estrogen receptors.

*Jatropha gossypifolia* is a traditional plant used for family planning [11]. It was found that this plant has a variety of phytochemical constituents, which have a multiplicity of pharmacological actions. The present preliminary phytochemical investigation on leaves extracts reveals the presence of carbohydrates, steroids, glycosides, flavonoids, alkaloids, and tannins in ethanolic extract. The ethanolic extract of *J. gossypifolia* revealed more significant estrogenic activity with the increase in uterine weight, as compared to control group of rats.

The results of the present study provide the evidence for the antifertility activity of *Jatropha gossypifolia* as claimed in the traditional use. The terpenoids, phytosterols, and flavonoids present in the extracts may be responsible for their activity. Further studies are going on in this laboratory to find out the active principals and the exact mechanism of action.

With these preliminary results, we can conclude that the ethanolic and aqueous extracts showed significant antifertility activity by means of potent estrogenic, anti-implantation, and early abortifacient activities in a dose-dependent manner. 

## Figures and Tables

**Table 1:** Effect of *Jatropha gossypifolia* leaves extracts on uterine weight of immature female rats.

Group	Extracts/drug	Dose (mg kg^−1^)	Uterine weight (mg)
I	Control (vehicle)	—	214.25 ± 22.79
II	Ethinyl estradiol (standard)	0.02	283.12 ± 23.50*
III	Ethanolic extract	200	323.25 ± 08.78***
IV	Ethanolic extract	400	351.57 ± 11.22**
V	Aqueous extract	200	283.12 ± 23.50*
VI	Aqueous extract	400	292.5 ± 10.30*

Values are mean ± SEM (*n* = 6). *P < 0.05, **P < 0.01, and ***P < 0.001 as compared to control group.

**Table 2:** Effect of *Jatropha gossypifolia *leaf extracts on anti-implantation and early abortifacient activity in rats (*x* ± *s*, *n* = 6).

Treatment/dose	% anti-implantation activity	% early abortifacient activity	% antifertility activity
Vehicle control	0	0	0
Ethanolic extract 200 mg kg^−1^, p.o.	24.35 ± 0.58	2.85 ± 0.59**	26.47 ± 0.65
Ethanolic extract 400 mg kg^−1^, p.o.	43.98 ± 0.40	5.71 ± 2.54***	74.27 ± 0.29
Aqueous extract 200 mg kg^−1^, p.o.	29.47 ± 0.39	3.03 ± 0.42**	33.31 ± 0.16
Aqueous extract 400 mg kg^−1^, p.o.	63.70 ± 0.34	10.08 ± 4.15***	46.78 ± 0.28

**P < 0.01, ***P < 0.001 versus vehicle control.
